# Effects of Different Feeding Regimes on Rumen Microbial Composition, Functional Potential, and Fermentation Characteristics of Longdong Goats (*Capra hircus*)

**DOI:** 10.3390/ani16101441

**Published:** 2026-05-08

**Authors:** Ke Wang, Junjie Hu, Ting Lu, Yong Zhang, Xingxu Zhao, Junxiang Yang

**Affiliations:** 1 College of Veterinary Medicine, Gansu Agricultural University, Lanzhou 730070, China; qydonkey123@163.com (K.W.); hujj@gsau.edu.cn (J.H.); asdlting@163.com (T.L.); zhangyong@gsau.edu.cn (Y.Z.); 2 Key Laboratory of Sheep & Goat Genetic Resources Evaluation and Utilization, Animal Husbandry and Veterinary Research Institute of Gansu Province, Ministry of Agriculture and Rural Affairs, Pingliang 744000, China

**Keywords:** *Capra hircus*, Longdong black goat, rumen microbiome, short-chain fatty acid

## Abstract

The rumen microbiota plays a key role in nutrient fermentation and short-chain fatty acid (SCFA) production in ruminants. However, the impacts of different feeding regimes on rumen microbial composition, functional potential, and metabolic outputs remain unclear. In this study, rumen fluid samples were collected from 12 Longdong goats (*Capra hircus*), which were divided into four groups based on feeding regime and coat color: housed white goats (n = 3), housed black goats (n = 3), grazing white goats (n = 3), and grazing black goats (n = 3). Samples were analyzed using high-throughput sequencing combined with functional annotation (KEGG and CAZy) and targeted SCFA profiling. Distinct differences in microbial community composition were observed primarily between feeding regimes, with enrichment of taxa related to carbohydrate degradation and fermentation. Functional analyses revealed significant shifts in metabolic pathways, particularly those associated with carbohydrate metabolism, energy production, and glycan biosynthesis. Several glycoside hydrolase and glycosyltransferase families showed differential abundances across groups. Consistently, SCFA concentrations varied significantly among feeding regimes. Correlation analyses further demonstrated strong associations between key microbial taxa, functional pathways, and specific SCFAs. Overall, these results indicate that feeding regime, rather than coat color, plays a dominant role in shaping rumen microbial structure, functional capacity, and fermentation characteristics, providing insights into microbial mechanisms underlying rumen metabolism and informing feeding strategy optimization.

## Simple Summary

The rumen microbiota plays an essential role in digesting plant-based diets in ruminants by fermenting feed components and producing short-chain fatty acids (SCFAs), which provide major energy sources for the host. This study aimed to investigate how feeding regimes influence the rumen microbial ecosystem in Longdong goats with different coat-color breeds. Rumen fluid samples were collected from black and white Longdong goats raised under different feeding conditions, including grazing and barn-feeding systems. High-throughput sequencing, together with KEGG and CAZy functional annotation and targeted SCFA analysis, was used to characterize microbial composition, functional potential, and metabolic outputs. The results showed that feeding conditions strongly shaped rumen microbial community structure and diversity. Grazing goats exhibited higher abundances of microorganisms and carbohydrate-active enzymes involved in plant polysaccharide degradation, accompanied by distinct SCFA profiles.

## 1. Introduction

Longdong goats (*Capra hircus*) are an important indigenous goat breed in China and are primarily distributed in the Ziwuling region of eastern Gansu Province, including surrounding semi-agricultural and semi-pastoral areas such as Huan County, Huachi County, and Heshui Counties. They are well adapted to the harsh, arid conditions of the Loess Plateau, with moderate body size, robust conformation, strong adaptability to roughage, and suitability for extensive or semi-intensive production systems [1]. Valued for meat, skin, and cashmere production, Longdong goats exhibit tender, flavorful meat and good fattening performance under grazing conditions [2], highlighting their economic potential. Their unique evolutionary background may also contribute to a resilient rumen microbiome capable of maintaining metabolic efficiency under varying feeding regimes.

The rumen is a specialized digestive organ in ruminants that provides an anaerobic and nutrient-rich environment for microbial fermentation [3,4]. A diverse consortium of microorganisms, including bacteria, archaea, protozoa, and fungi, cooperatively degrades complex plant-derived polymers such as cellulose, hemicellulose, and starch [5]. Through these coordinated metabolic activities, rumen microorganisms convert dietary substrates into fermentation end products that accumulate in rumen fluid [6], among which short-chain fatty acids (SCFAs) represent the principal products and play a central role in nutrient utilization and energy metabolism in ruminants [7,8]. These metabolic activities directly shape the rumen fluid metabolome and determine both the composition and yield of SCFAs, thereby influencing feed utilization efficiency and key production traits such as growth performance, body weight gain, and fat deposition [9,10,11,12]. Consequently, variations in rumen microbial metabolism and fermentation patterns are closely associated with differences in host nutritional status and productive performance [13]. Previous studies in ruminants (e.g., cattle and sheep) have demonstrated that feeding regime is a major factor shaping rumen microbial composition and fermentation patterns [14,15]. However, most studies have primarily focused on dietary effects, while host-related traits have received comparatively less attention. As a visible phenotypic trait, coat color may be associated with underlying genetic variation and can be considered as a host-related factor when examining variation in rumen microbial communities.

However, despite the economic importance of Longdong goats, the metabolic characteristics of rumen fluid and their associations with short-chain fatty acid profiles and microbial functions remain insufficiently understood. This study aimed to (1) evaluate the effects of host-related phenotypic traits (white versus black Longdong goats) and feeding regime (barn- versus pasture-feeding) on rumen microbial communities and fermentation patterns, and (2) investigate the relationships between microbial composition, functional potential, and SCFA production. Variation in rumen microbial composition, functional potential, and SCFA production may be associated with feeding regime and host-related phenotypic traits. By integrating high-throughput metagenomic sequencing with rumen fluid metabolomics and SCFA analyses, this study provides a comprehensive multi-omics characterization of the rumen ecosystem in Longdong goats, offering new insights into the associations between microbial composition, functional potential, and feed utilization in this indigenous breed.

## 2. Materials and Methods

### 2.1. Study Area and Experimental Design

The experiment was conducted from 25 May to 26 October 2024 at a cooperative located in Chedao Town, Huan County, one of the main production areas of Longdong goats, Gansu Province, China (36.33° N, 106.73° E). The study area is situated in the northwestern part of Huan County, where the terrain is characterized by hills and gullies, with an average altitude of 1426 m. The region has a mean annual temperature of 7.5 °C, an average frost-free period of approximately 120 days, and a mean annual precipitation of 425.4 mm.

A total of 12 experimental goats were selected from a local meat goat breeding cooperative in Huan County, with a body weight of 20–25 kg and an estimated age of 6–8 months. Sex was not recorded and was not considered as a variable in this study. The experiment followed a 2 × 2 factorial design, with feeding regime (grazing vs. barn-fed) and coat color (white vs. black) as fixed factors. Animals were randomly assigned to one of four experimental groups (n = 3 per group): grazing white goats (WF), barn-fed white goats (WB), grazing black goats (BF), and barn-fed black goats (BB). Goats in the barn-fed groups were fed a self-formulated diet consisting mainly of maize, peas, and wheat bran as concentrate, and maize straw supplemented with small amounts of alfalfa and oat hay as roughage. The daily concentrate intake was approximately 450–500 g per animal.

Goats in the barn-fed group were fully confined without access to grazing. Goats in the grazing plus supplementary feeding group grazed freely throughout the day, consuming natural pasture grasses and tree leaves. The main forage species in the grazing area included *Stipa* spp., *Bothriochloa ischaemum*, *Leymus secalinus*, *Artemisia* spp., *Thymus* spp., *Achnatherum* spp., and fallen leaves of *Robinia pseudoacacia*. In addition, a small amount of maize grain was supplied daily as a supplement. Animals were maintained under their respective feeding conditions for 5 months prior to sample collection. The diet composition was described based on available feed ingredients under field conditions, and detailed nutrient composition (e.g., crude protein, NDF, ADF, and metabolizable energy) was not available. Therefore, the observed differences are interpreted primarily in the context of feeding management rather than specific dietary components.

### 2.2. Sampling and Sequencing

Rumen fluid was collected immediately after slaughter from slaughtered goats using a 50 mL sterile syringe from the ventral sac of the rumen. The collected fluid was filtered through six layers of sterile gauze, and approximately 100 mL of filtrate was transferred into a sterile beaker. Samples were temporarily stored on ice and transported to the laboratory within 10 h, then stored in liquid nitrogen (−196 °C) for further analysis to minimize post-mortem changes.

Total microbial DNA was extracted using the QIAamp^®^ Fast DNA Stool Mini Kit (Qiagen, Hilden, Germany). DNA concentration and integrity were assessed using a NanoDrop 2000 spectrophotometer (Thermo Fisher Scientific, Waltham, MA, USA) and agarose gel electrophoresis. DNA fragmentation was performed using an S220 Focused-ultrasonicator (Covaris, Woburn, MA, USA), followed by purification with Agencourt AMPure XP beads (Beckman Coulter, Brea, CA, USA). Sequencing libraries were prepared using the TruSeq Nano DNA LT Sample Preparation Kit (Illumina, San Diego, CA, USA). Library construction, sequencing, and data analysis were conducted by OE Biotech Co., Ltd. (Shanghai, China).

The sequencing libraries were sequenced on an Illumina NovaSeq 6000 platform to generate 150 bp paired-end reads. Raw reads were quality-filtered and adapter-trimmed using fastp (v0.20.1) (parameter: -a auto --adapter_sequence_r2 auto --detect_adapter_for_pe -f 0 -F 0 --cut_front --cut_tail --n_base_limit 5 --cut_window_size 4 --cut_mean_quality 20 --length_required 75 --qualified_quality_phred 15) [16], and reads containing ambiguous bases were removed. Host-derived sequences were identified and discarded by mapping clean reads to the host genome using BBMap (v38.93-0) (parameter: nodisk k = 13 minid = 0.90 usemodulo = t fast = t noheader = t notags = t) [17].

Valid reads were assembled de novo using MEGAHIT (v1.2.9) (parameter: --k-list 55 --min-contig-len 200) [18]. Open reading frames (ORFs) were predicted from assembled scaffolds using Prodigal (v2.6.3) (parameter: -p meta -f gff) [19] and translated into amino acid sequences. A non-redundant gene catalog was constructed across all samples using MMSeqs2 (v13.45111) (parameter: -c 0.9 --min-seq-id 0.95 --cov-mode 1 -v 1 --cluster-mode 2 --max-seq-len 131070) [20] with clustering thresholds of 95% sequence identity and 90% coverage, and the longest sequence in each cluster was selected as the representative gene. Gene abundance was quantified by mapping clean reads to the non-redundant gene catalog using Salmon (v1.8.0) (parameter: -l IU --meta –validateMappings) [21] with 95% identity.

Taxonomic annotation was performed based on the NR database, and species abundance was calculated by summing the abundances of genes assigned to each taxon. Taxonomic profiles were generated at the domain, kingdom, phylum, class, order, family, genus, and species levels. Functional annotation of representative gene sequences was conducted using DIAMOND (v2.1.9) (parameter: -k 10 --fast --salltitles --outfmt 5) [22] against the NR, KEGG, COG, Swiss-Prot, and GO databases with an e-value threshold of 1 × 10^−5^. Carbohydrate-active enzymes were annotated using the CAZy database with HMMER hmmscan (v3.1), and CAZyme abundance was calculated based on the summed abundance of corresponding genes. Multivariate analyses, including PCA, PCoA, and NMDS, were performed using R (v4.1.2). Differential abundance analyses were conducted using ANOVA, Kruskal–Wallis, Student’s *t*-test, or Wilcoxon test as appropriate, and Linear discriminant analysis effect size (LEfSe) was applied to identify differentially abundant taxa or functional features. LEfSe analysis was performed with a significance threshold of *p* < 0.05 and an LDA score cutoff of 2.0 (or 3.0).

### 2.3. LC-MS/MS Analysis

Short-chain fatty acids (SCFAs) in rumen fluid were quantitatively analyzed using a targeted LC–MS/MS method. Rumen fluid samples were thawed on ice and centrifuged at 12,000× *g* for 10 min at 4 °C to remove particulate matter. Short-chain fatty acids (SCFAs), including acetic acid, propionic acid, butyric acid, pentanoic acid, hexanoic acid, isobutyric acid, isovaleric acid, lactic acid, malonic acid, succinic acid, glutaric acid, and 2,3-dihydroxy-3-methylbutanoic acid, were quantified using liquid chromatography–tandem mass spectrometry (LC–MS/MS). Authentic standards (purity ≥ 99%) were purchased from commercial suppliers as listed in Table S1. Stable isotope–labeled [^2^H_9_]-pentanoic acid was used as the internal standard for quantification. Analytes were detected in negative electrospray ionization (ESI^−^) mode using multiple reaction monitoring (MRM). Compound-specific precursor/product ion transitions (Q1/Q3) and retention times were optimized using individual standards and are summarized in Table S1. Quantification was performed based on peak area ratios of each analyte to the internal standard.

### 2.4. Statistical Analysis

The data were analyzed using the free online platform Oebiotech Cloud (https://cloud.oebiotech.com/). Principal component analysis (PCA) and orthogonal partial least squares discriminant analysis (OPLS-DA) were performed using the R package “ropls” (Version 1.6.2), with sevenfold cross-validation to evaluate model stability. Differences among groups were assessed using one-way analysis of variance (ANOVA) followed by Tukey’s multiple comparison test, or the Kruskal–Wallis test when data did not meet the assumptions of normality. For comparisons between two groups, the Wilcoxon rank-sum test was applied. A *p*-value < 0.05 was considered statistically significant.

## 3. Results

### 3.1. Microbial Composition of Rumen Fluid

A total of 12 metagenomic libraries were sequenced, yielding 77.35–82.25 million clean reads per sample (99.95–99.97%). Clean bases ranged from 11.53 to 12.30 Gb, with GC content of 44.78–48.91% and high quality (Q20: 98.77–99.08%; Q30: 95.81–96.87%) (Table S2). These results indicate that the sequencing data were of high quality and suitable for subsequent metagenomic assembly and downstream functional analyses.

**Figure 1:**
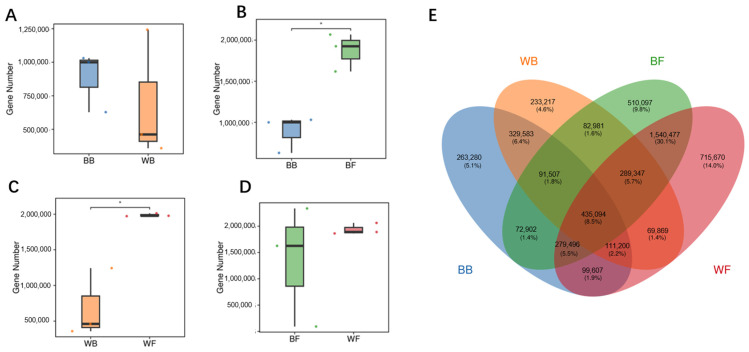
Gene number comparison and gene sharing among experimental groups. (**A**–**D**) Boxplots showing comparisons of predicted gene numbers between paired groups, (**E**) Venn diagram illustrating shared and unique genes among BB, WB, BF, and WF groups. Statistical significance between groups is indicated by asterisks (* *p* < 0.05). Note: WF, grazing white goats; WB, barn-fed white goats; BF, grazing black goats; BB, barn-fed black goats.

High-quality clean reads from all samples were assembled to construct a non-redundant gene catalog. A substantial number of predicted genes were obtained from each experimental group, with clear differences observed among groups. Specifically, the BB group harbored approximately 0.8–1.0 million predicted genes, whereas the WB group contained a markedly lower number of genes, mainly ranging from 0.4 to 0.6 million (Figure 1A). In contrast, the grazing groups exhibited significantly higher gene numbers, generally exceeding 1.8 million predicted genes per group (Figure 1B–D). Pairwise comparisons further revealed significant differences in gene numbers between black goat groups, as well as between white goat groups, indicating distinct genomic complexity among experimental conditions. No pronounced difference was detected between grazing black goats and grazing white goats, both of which maintained similarly high gene numbers. Venn diagram analysis was performed to examine the overlap and specificity of predicted genes among the four experimental groups (Figure 1E). A substantial number of genes were shared by all groups, representing the common gene set of the rumen microbiome. In addition, each group harbored a considerable number of unique genes that were not detected in the other groups, indicating clear differences in gene composition among four groups.

**Figure 2:**
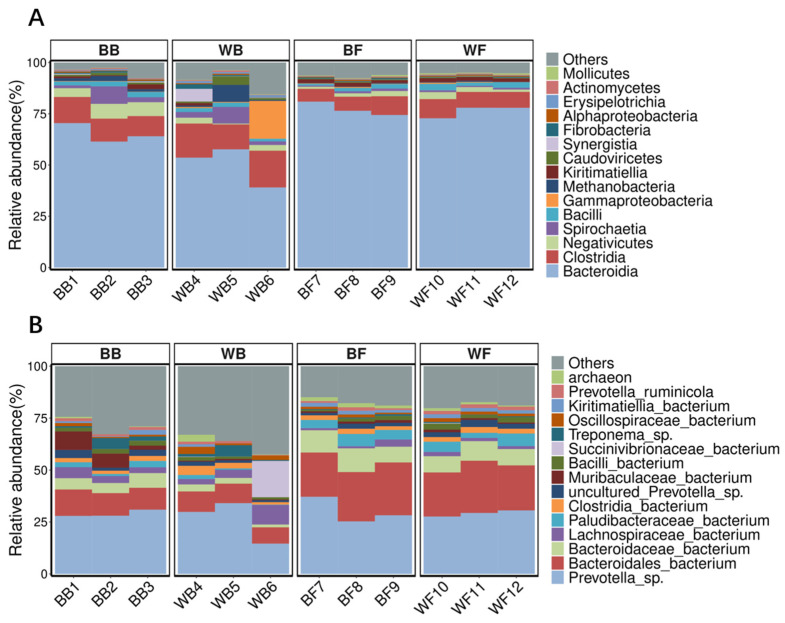
Taxonomic composition of rumen fluid microbiota in black and white goat under different feeding systems. (**A**) Relative abundance of microbial taxa at the class level. (**B**) Relative abundance of dominant taxa at the genus level.

At both the class and genus levels, distinct differences in microbial community composition were observed between black goat groups and white goat groups (Figure 2). At the class level, Bacteroidia and Clostridia dominated all samples, while their relative abundances differed between the two goat phenotypic traits. The B groups generally exhibited a higher and more stable proportion of Bacteroidia, whereas the W groups showed greater variability and a relatively higher contribution of other bacterial classes. At the genus level, *Prevotella* sp. and *Bacteroidales bacterium* were the predominant taxa across all groups, but their relative abundances differed noticeably between black and white goat. In comparison with the black goat groups, the white goat groups displayed increased proportions of several minor taxa and a higher fraction of unclassified or low-abundance genera, indicating differences in community structure between the two kind of goat phenotypic traits.

When comparing feeding systems, distinct differences were also observed between Barn (B) and farm (F) groups (Figure 2). At the class level, the grazing groups (BF and WF) were characterized by a higher dominance of Bacteroidia and a more consistent community structure among replicates, whereas the barn groups (BB and WB) showed relatively higher proportions of Clostridia and greater inter-individual variation. At the genus level, grazing groups exhibited increased relative abundances of *Prevotella* sp. and other *Bacteroidales*-related taxa, while stall-fed groups showed more heterogeneous genus-level profiles, with higher contributions from diverse low-abundance taxa. These patterns indicate that feeding system exerts a strong influence on rumen microbial community composition, potentially shaping the overall structure of the rumen microbiota.

### 3.2. Alpha Diversity and Beta Diversity of Rumen Microbial Communities

**Figure 3:**
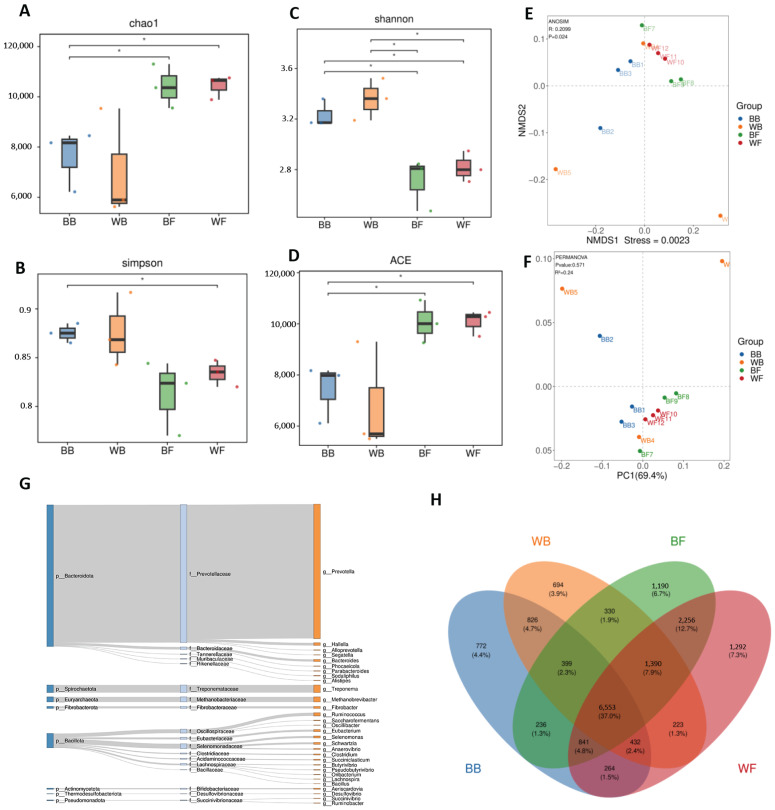
Alpha and beta diversity of rumen microbial communities in four groups (BB, WB, BF and WF). (**A**) Chao1 index, (**B**) Simpson index, (**C**) Shannon index, and (**D**) ACE index showing microbial richness and diversity across groups. Boxes represent the interquartile range with the median indicated, and whiskers denote the minimum and maximum values. Significant differences between groups are indicated by asterisks (* *p* < 0.05). (**E**) Non-metric multidimensional scaling (NMDS) based on Bray–Curtis dissimilarity. (**F**) Principal coordinate analysis (PCoA) showing differences in microbial community composition among groups. (**G**) Sankey diagram illustrating the taxonomic flow of microbial species from phylum to family and genus levels across all samples. (**H**) Venn diagram showing the number of shared and unique microbial species among BB, WB, BF, and WF groups.

Alpha diversity indices revealed significant differences in microbial richness and diversity among the four rumen fluid groups (BB, WB, BF and WF) (Figure 3A–D). The Chao1 and ACE indices, which reflect community richness, showed that grazing groups had significantly higher values than barn-fed groups (Figure 3A,D), indicating a greater number of microbial taxa in these two groups. Among them, grazing white goats exhibited the highest richness, followed by grazing black goats, whereas barn-fed white goats showed the lowest richness. The Shannon index, which considers both richness and evenness, was significantly higher in barn-fed groups than in grazing groups (Figure 3C), suggesting that although grazing groups harbored more taxa, their communities were dominated by fewer highly abundant species, leading to reduced evenness. Similarly, the Simpson index showed that barn-fed groups had significantly higher diversity than grazing groups (Figure 3B), further supporting that microbial communities in grazing groups were less even and more strongly structured by dominant taxa. Overall, these results indicate that grazing groups were characterized by higher species richness but lower community evenness, whereas barn-fed groups maintained more evenly distributed microbial communities. Beta diversity analysis based on NMDS and PCoA demonstrated clear separation of microbial communities among the four groups (Figure 3E,F). The NMDS ordination showed that samples from four groups formed distinct clusters with minimal overlap (Stress = 0.0023), indicating pronounced differences in microbial community composition among groups (Figure 3E). Consistently, the PCoA analysis also revealed that samples from different groups were well separated along the first two principal coordinates, which together explained a large proportion of the total variation (Figure 3F). Grazing groups clustered separately from barn-fed groups, suggesting that these two groups harbored distinct microbial assemblages. Together, the beta diversity results indicate that the microbial community structures differed significantly among the four rumen fluid groups, reflecting strong group-specific microbial configuration.

### 3.3. Differential Microbial Taxa Between Groups

**Figure 4:**
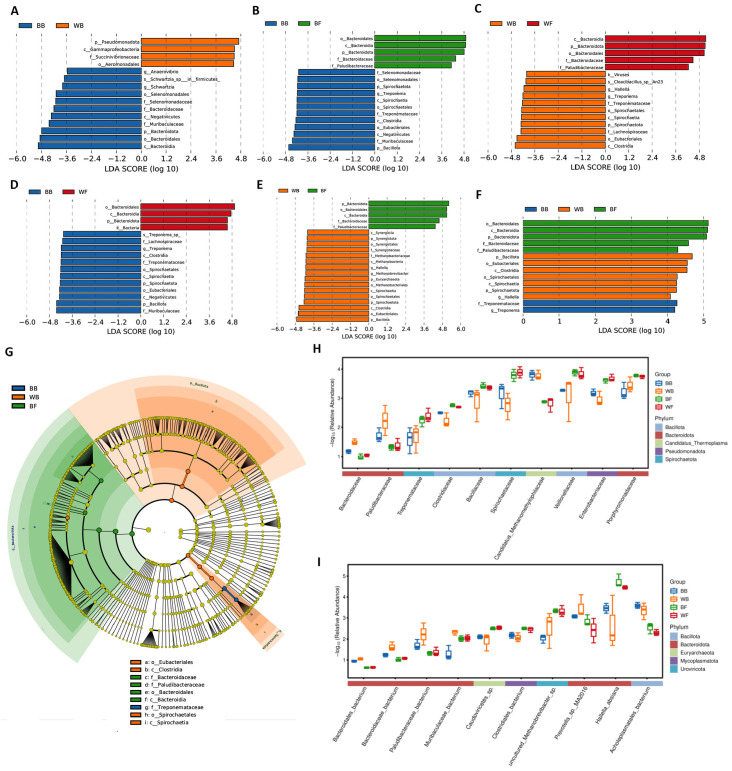
Phylogenetic structure and abundance patterns of LEfSe-identified microbial biomarkers. (**A**–**F**) The boxplots show the relative abundance of representative LEfSe-identified taxa at the family level (**middle**) and species level (**right**) across BB, WB, BF, and WF. Boxes represent the interquartile range, center lines indicate medians, and whiskers denote the full range of values, with individual points representing samples. (**G**) The LEfSe cladogram depicts the taxonomic hierarchy of microbial lineages that are differentially enriched among the four groups, with colored branches indicating taxa significantly associated with each group. The concentric rings represent taxonomic levels from phylum (**inner**) to species (**outer**). (**H**,**I**) Boxplots showing the relative abundance of representative differential taxa across groups.

Linear discriminant analysis effect size (LEfSe) analysis revealed clear microbial biomarkers associated with both goat coat color (black vs. white) and feeding regime (grazing vs. barn-feeding) (Figure 4). Across all pairwise and multi-group comparisons, two contrasting microbial assemblages were consistently observed, corresponding to fiber-degrading and readily fermentable substrate-utilizing communities. In the comparison between BB and WB, taxa affiliated with Bacteroidota were significantly enriched in WB, including Bacteroidia, Bacteroidales, Bacteroidaceae, Paludibacteraceae, as well as members of Gammaproteobacteria such as Aeromonadales and Succinvibrionaceae. These taxa are typically associated with the degradation of starches, proteins, and other easily fermentable substrates. In contrast, barn-fed black goats showed significant enrichment of taxa related to fiber degradation and anaerobic fermentation, including Clostridia, Negativicutes, Muribaculaceae, Selenomonadaceae, and the spirochaetal genus Schwartzia and Anaerovibrio, indicating a microbial community more adapted to structural carbohydrate utilization.

When gazing black goats were compared with barn-fed black goats, gazing black goats was strongly enriched in Bacteroidota-related lineages, including Bacteroidales, Bacteroidia, Bacteroidota, Bacteroidaceae, and Paludibacteraceae, whereas BB showed higher abundances of Bacillota, Muribaculaceae, Negativicutes, Eubacteriales, Clostridia, and Spirochaetota (including Treponema), reflecting a shift toward fiber-associated microbial guilds under grazing conditions. Similarly, in the WB vs. BF comparison, BF was again characterized by enrichment of Bacteroidota and Bacteroidales, while WB was associated with Clostridia, Spirochaetota, Eubacteriales, Methanobacteriales, and Methanobrevibacter, indicating that feeding regime strongly modulates the balance between carbohydrate-fermenting and fiber-degrading consortia. White goat under stall-feeding (WF) exhibited significantly higher abundances of Bacteroidota, Bacteroidia, Bacteroidales, and Bacteroidaceae, consistent with an increased capacity for the utilization of readily fermentable substrates. In contrast, WB was enriched in Treponema, Spirochaetota, Clostridia, Lachnospiraceae, and Eubacteriales, further supporting the distinction between fiber-oriented and fermentative microbial strategies. In the multi-group LEfSe analysis (BB, WB, BF), grazing black goats showed the strongest enrichment of Bacteroidota-associated taxa, whereas barn-fed white goats were characterized by higher abundances of Bacillota, Clostridia, Spirochaetales, and Spirochaetota. Barn-fed black goats were uniquely associated with Treponema and Treponemataceae, highlighting their specialization in fiber degradation. Collectively, LEfSe analysis demonstrated that grazing and black-coated goats were preferentially associated with fiber-degrading and short-chain fatty acid-producing microbial taxa, such as Treponema, Clostridia, and Lachnospiraceae, whereas stall-fed and white-coated goats harbored communities enriched in fast-fermenting taxa, including Bacteroidales, Prevotellaceae, and Paludibacteraceae. These results indicate that both coat color and feeding regime jointly shape distinct rumen microbial strategies, potentially influencing fiber utilization efficiency and fermentation patterns.

To further validate the taxa identified by LEfSe, the relative abundances of representative biomarkers at both family and species levels were visualized using boxplots (Figure 4A–F). At the family level, clear group-dependent abundance patterns were observed among several dominant rumen microbial lineages. Members of Bacteroidaceae and Porphyromonadaceae showed higher relative abundance in the white goat groups compared with black goat groups, whereas Ruminococcaceae and Clostridiaceae tended to be more abundant in the grazing groups. In addition, Spirochaetaceae and *Candidatus* Methanomethylophilaceae, which are associated with hydrogen metabolism and methanogenesis, displayed elevated abundances particularly in BF and WF, consistent with their enrichment detected by LEfSe. These results indicate that dietary treatments reshaped not only the overall community structure but also the abundance of key functional microbial families.

At the species level, several dominant taxa exhibited consistent shifts across groups. Species affiliated with *Prevotella* (e.g., *Prevotella* sp. MA2016) and Bacteroidaceae bacterium were relatively more abundant in white goat groups, whereas *Methanobrevibacter* sp. and *Clostridiales bacterium* showed higher abundance in grazing condition. Notably, *Halieella absiana* and *Acholeplasmatales bacterium* also displayed clear group-specific enrichment patterns, supporting the LEfSe-based identification of these taxa as important contributors to the observed community differentiation.

### 3.4. Functional Potential of Rumen Microbiota Revealed by CAZy and KEGG Analyses

CAZy profiling revealed that grazing groups (BF and WF) harbored significantly higher abundances of carbohydrate-active enzyme families, including glycoside hydrolases (GH), glycosyltransferases (GT), carbohydrate esterases (CE), polysaccharide lyases (PL), and carbohydrate-binding modules (CBM), compared with barn-fed groups (BB and WB). Among them, BF exhibited the highest overall CAZy abundance. These patterns indicate an enhanced capacity for degradation of complex plant polysaccharides in grazing goat. KEGG-based LEfSe analysis revealed distinct functional profiles among groups. WF was enriched in pathways related to central carbon metabolism, including glycolysis, the TCA cycle, carbon fixation, and starch and sucrose metabolism, indicating elevated energy and carbohydrate metabolism. Grazing black goats showed enrichment in lipid metabolism and host-interaction-related pathways such as sphingolipid metabolism, PPAR signaling, and ABC transporters. In contrast, barn-fed white goats were characterized by pathways involved in motility and environmental sensing, including flagellar assembly and chemotaxis, while BB was mainly enriched in two-component systems and antibiotic-related pathways.

**Figure 5:**
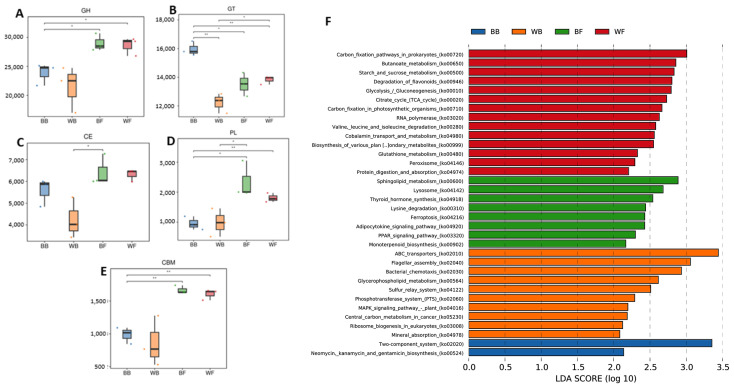
Functional differentiation of rumen microbiota among BB, WB, BF and WF groups based on CAZy and KEGG analyses. (**A**–**E**) Boxplots showing the relative abundance of the five major CAZy families: glycoside hydrolases (GH), glycosyltransferases (GT), carbohydrate esterases (CE), polysaccharide lyases (PL), and carbohydrate-binding modules (CBM) across the four groups. (**F**) KEGG pathway enrichment based on LEfSe analysis. Bars represent LDA scores (log10), indicating pathways significantly enriched in each group. Different colors denote the four groups: BB (blue), WB (orange), BF (green), and WF (red). Only pathways with LDA score above the significance threshold are shown. Statistical significance between groups is indicated by asterisks (* *p* < 0.05, ** *p* < 0.01).

To further resolve which carbohydrate-active enzyme families contributed to the functional divergence among groups, representative glycosyltransferase (GT) and glycoside hydrolase (GH) families were compared in detail (Figure 5).

**Figure 6:**
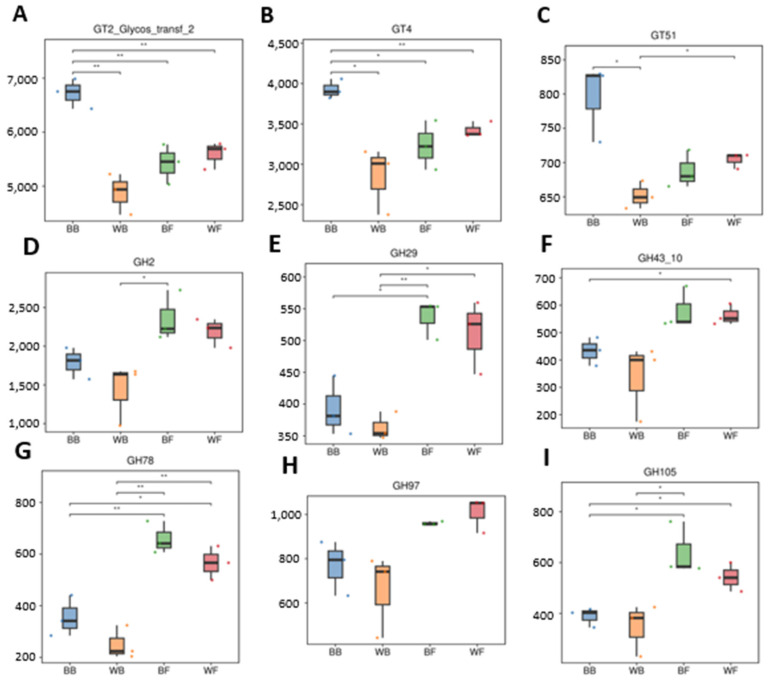
Differential abundance of representative CAZy families across BB, WB, BF, and WF groups. Boxplots depict the relative abundances of three glycosyltransferase families ((**A**–**C**): GT2, GT4, GT51) and six glycoside hydrolase families ((**D**–**I**): GH2, GH29, GH43_10, GH78, GH97, GH105) among the four groups. These CAZy families are associated with the synthesis and degradation of plant-derived polysaccharides. Statistical significance between groups is indicated by asterisks (* *p* < 0.05, ** *p* < 0.01). Colors correspond to BB (blue), WB (orange), BF (green), and WF (red).

Among glycosyltransferases, GT2, GT4 and GT51 showed consistent and significant differences among groups (Figure 5A–C). All three GT families exhibited their lowest abundance in barn-fed white goats, whereas grazing black goats and grazing white goats showed significantly higher levels, indicating enhanced glycan synthesis and cell wall-associated carbohydrate metabolism in grazing animals. Notably, barn-fed black goats maintained relatively high GT2, GT4 and GT51 levels compared with WB, suggesting a phenotype-related effect even under barn feeding. For glycoside hydrolases, strong enrichment of plant polysaccharide-degrading families was observed in grazing groups (BF and WF) (Figure 6D–I). GH2 and GH29, which are involved in hemicellulose and oligosaccharide degradation, were significantly more abundant in grazing groups (BF and WF) than in barn-fed groups (BB and WB). Similarly, GH43_10, GH78, GH97 and GH105, all associated with xylan, arabinan, pectin, and complex plant carbohydrate degradation, showed marked increases in grazing goat, with grazing black goats generally exhibiting the highest values. In contrast, barn-fed white goats consistently displayed the lowest abundance across most GH families, indicating a reduced capacity for fiber and complex polysaccharide utilization under barn feeding in white goat. Although barn-fed black goats showed higher GH levels than barn-fed white goats, they remained significantly lower than those observed in grazing groups, suggesting that feeding regime exerts a stronger influence on CAZyme composition than host-related phenotypic traits.

### 3.5. Short-Chain Fatty Acid (SCFA) Profiles

**Figure 7:**
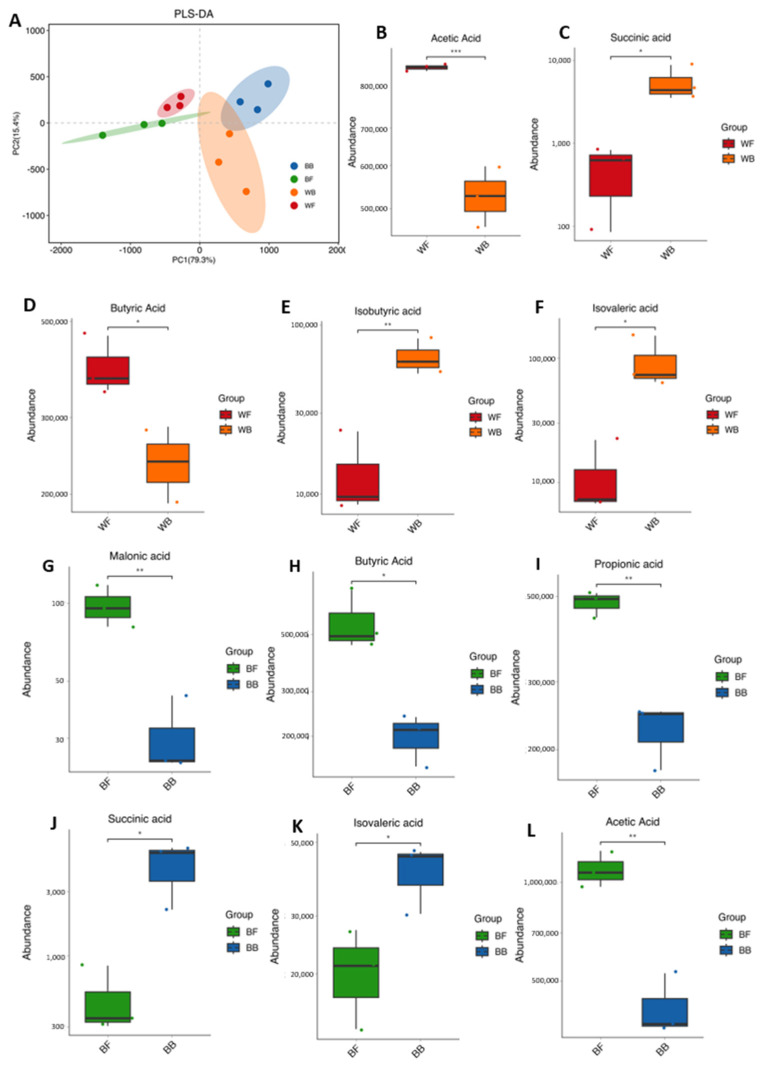
Partial least squares discriminant analysis (PLS–DA) of rumen microbial communities and short–chain fatty acid (SCFA) profiles across four groups (BB, WB, BF, WF). (**A**) PLS–DA score plot showing separation among the four groups based on rumen microbial composition. Percentages on axes indicate the proportion of variance explained by each component. (**B**–**F**) Boxplots of SCFA abundances comparing grazing white (WF) and barn-fed white (WB) goats: (**B**) acetic acid, (**C**) succinic acid, (**D**) butyric acid, (**E**) isobutyric acid, (**F**) isovaleric acid. (**G**–**L**) Boxplots of SCFA abundances comparing grazing black (BF) and barn-fed black (BB) goats: (**G**) malonic acid, (**H**) butyric acid, (**I**) propionic acid, (**J**) succinic acid, (**K**) isovaleric acid, (**L**) acetic acid. Colors represent WF (red), WB (orange), BF (green), and BB (blue). Statistical significance between groups is indicated by asterisks (* *p* < 0.05, ** *p* < 0.01, *** *p* < 0.005).

PLS-DA based on short-chain fatty acid (SCFA) profiles demonstrated a clear separation among four groups, indicating pronounced differences in ruminal fermentation patterns across feeding regimes (Figure 7A).

In the comparison between white goat groups, razing white goats showed significantly higher concentrations of major fermentation products, including acetic acid, butyric acid, and propionic acid. In contrast, succinic acid, isobutyric acid, and isovaleric acid were significantly enriched in barn-fed white goats relative to grazing white goats (Figure 7B–F). Similarly, significant differences in SCFA composition were observed between black goat groups (BF and BB). Compared with BB, BF exhibited significantly higher levels of malonic acid, butyric acid, propionic acid, and acetic acid. Conversely, succinic acid and isovaleric acid were significantly more abundant in BB than in BF (Figure 7G–L).

Overall, these results demonstrate that feeding regimes significantly alter both the concentration and composition of ruminal short-chain fatty acids, with consistent enrichment of major SCFAs in grazing groups and higher levels of certain intermediate and branched-chain acids in barn-fed groups.

## 4. Discussion

### 4.1. Feeding Regime Reshapes Ruminal Microbial Diversity and Community Structure

The observed differences in alpha diversity indices among groups were primarily associated with feeding regimes rather than phenotype-related differences. Although significant differences were observed between barn-fed black goats and grazing white goats, comparisons within the same feeding regime indicated that phenotype effects were less pronounced than those of feeding management. The farming group exhibited significantly higher Chao1 and ACE indices, indicating increased microbial richness compared with the barn-fed group. In contrast, Shannon and Simpson indices were higher in the barn-fed group, suggesting greater community evenness and reduced dominance of rare taxa. In parallel, beta-diversity analyses based on NMDS ordination showed clear separation among groups, further suggesting that microbial community composition differed substantially under different feeding conditions. The observed clustering patterns indicated that samples within the same feeding regime tended to group together, whereas differences were observed between grazing and barn-fed systems. These results suggest that grazing conditions may be associated with a broader range of microbial tax [15,23], whereas barn feeding may contribute to a more even and stable microbial community structure. This observation is consistent with previous reports that diet composition and feeding management are important factors influencing rumen microbial ecology [11,24,25]. These results indicate that feeding strategies are the primary drivers of rumen microbial diversity in Longdong goats.

### 4.2. Distinct Microbial Taxa Associated with Different Feeding Regimes

The observed differences in rumen microbial community composition between white- and black-coated Longdong goats suggest that host-related factors at the phenotype level may contribute to shaping rumen microbiota structure. Similar phenotypic trait-associated microbial patterns have been reported in other ruminants, including cattle [26], sheep [27,28], and goats [29], even under comparable feeding conditions, suggesting the role of host genetics and physiological traits in modulating microbial assembly. Across multiple comparisons, black goats—particularly under grazing conditions—showed consistent enrichment of fiber-degrading and anaerobic fermentative lineages, including Clostridia- and Negativicutes-related taxa, as well as members of Muribaculaceae, Selenomonadaceae, Treponema, and Anaerovibrio. These microbes are known for their roles in structural carbohydrate degradation and short-chain fatty acid production [30,31,32], indicating a rumen microbiota in black goats that is more specialized for fibrous feed utilization. In contrast, white goats showed higher relative abundances of Bacteroidales and its affiliated families (e.g., Bacteroidaceae, Paludibacteraceae, and Prevotellaceae), which are typically associated with the degradation of readily fermentable substrates, including starches, proteins, and soluble polysaccharides [33,34]. However, given that this study is based on compositional and association analyses, these differences should be interpreted as observed patterns rather than direct evidence of functional specialization. Overall, the results indicate that microbial community composition varies between phenotypic groups, although the underlying mechanisms remain to be further investigated.

Feeding regime emerged as a dominant factor shaping rumen microbial community structure, as evidenced by consistent shifts in key microbial taxa across feeding comparisons. Grazing conditions favored the enrichment of microbial groups functionally associated with plant cell wall degradation, including cellulolytic and hemicellulolytic bacteria commonly observed in ruminants consuming high-fiber diets, highlighting a conserved microbial response to pasture-based feeding systems. Similar grazing-related microbial shifts have been widely reported in other ruminants, such as cattle [35,36,37] and sheep [38], where pasture-based feeding systems consistently promote the proliferation of cellulolytic and hemicellulolytic bacteria, reflecting a conserved microbial response to high-fiber diets across ruminant species.

Conversely, stall-feeding promoted the enrichment of Bacteroidota-related taxa involved in the utilization of readily fermentable substrates, which may indicate a potential for metabolic versatility and rapid growth under non-structural carbohydrate-rich diets. Similar patterns have been reported in stall-fed cattle [39] and sheep [40,41]. The repeated observation of these patterns across multiple pairwise and multi-group LEfSe comparisons supports feeding management as a key ecological driver modulating the balance between fiber-degrading and fast-fermenting microbial consortia in the rumen.

Notably, multiple taxa within Bacteroidota, Firmicutes (Bacillota), and Spirochaetota exhibited significant enrichment in specific groups, as indicated by high LDA scores. These patterns were consistently supported by relative abundance comparisons at both the family and species levels, showing concordance between LEfSe-identified biomarkers and abundance-based analyses. Together, these results underscore the robustness of the detected microbial signatures and indicate that feeding regime selectively favors distinct microbial assemblages [42,43,44], reflecting differences in potential substrate utilization and ecological niches within the rumen [44,45]. Beyond individual pairwise comparisons, LEfSe analysis revealed recurrent functional patterns across groups. Irrespective of phenotype or feeding regime, rumen microbiota consistently organized into two contrasting functional assemblages: fiber-degrading communities adapted to complex plant polysaccharides [46,47] and fast-fermenting communities specialized for readily available substrates [48,49]. This functional dichotomy was repeatedly observed across multiple comparisons, suggesting a conserved ecological response of the rumen microbiome to dietary substrate availability [42]. Notably, feeding regime emerged as the primary driver shaping these functional strategies [50,51], while phenotype-related factors further modulated microbial composition within the same feeding context.

### 4.3. Effects of Feeding Regimes on Microbial Functional Potential and SCFA Production

Beyond taxonomic composition, functional profiling revealed pronounced differences in microbial metabolic potential across feeding regimes. Differences in the abundance of these CAZy families can suggest reflect shifts in microbial carbohydrate metabolism in response to dietary changes [52,53]. GH families associated with polysaccharide degradation and GT families involved in carbohydrate biosynthesis exhibited higher abundances in grazing-related groups, indicating a greater predicted capacity for complex carbohydrate utilization under forage-based diets [54,55]. Consistently, KEGG pathway analysis identified multiple metabolic pathways with significantly different enrichment patterns, as revealed by LEfSe. Pathways related to carbohydrate metabolism, energy production, and secondary metabolite biosynthesis were differentially represented among feeding regimes, reflecting differences in predicted functional potential of the rumen microbiota to distinct nutritional inputs and feeding management [56,57].

Short-chain fatty acids (SCFAs), the main end products of ruminal microbial fermentation, were further analyzed to assess whether taxonomic and functional shifts were accompanied by changes in fermentation outcomes [58]. Feeding regimes can shape rumen SCFA profiles, potentially reflecting the metabolic output of the microbial community [42]. Major SCFAs, such as acetic, propionic, and butyric acids, are closely linked to the degradation of dietary carbohydrates and proteins [59], while minor SCFAs, including succinic and isovaleric acids, may indicate alternative fermentation pathways [60]. Variations in SCFA composition are generally consistent with shifts in microbial community structure and functional potential, suggesting a potential link between diet, microbial composition, and fermentation outcomes [61,62]. Although direct causal relationships are difficult to establish, the alignment between microbial, functional, and metabolic profiles supports a potentially integrative response of the rumen ecosystem to different feeding regimes [43].

A limitation of this study is the relatively small sample size, which may limit the statistical power, particularly for the high-dimensional metagenomic and functional data involving multiple comparisons. Future work with larger cohorts is warranted to validate these microbial and functional patterns.

## 5. Conclusions

Feeding regime is the primary driver of rumen microbial community composition and functional capacity in Longdong goats, whereas phenotype differences play a minor role. Grazing goats harbored higher abundances of carbohydrate-degrading microbes and glycoside hydrolase families, which were associated with enhanced short-chain fatty acid production, particularly acetate, propionate, and butyrate. Functional pathway analyses further indicated that carbohydrate metabolism, energy production, and glycan biosynthesis were strongly modulated by diet. These findings reveal a clear link between microbial community structure, functional potential, and metabolic output in response to feeding strategies. Understanding these interactions provides a mechanistic basis for optimizing feeding management to improve nutrient utilization, animal performance, and sustainable goat production.

## Data Availability

The raw data used in this work were deposited at China National Center for Bioinformation (CNCB), BioProject: PRJCA057857.

## Supplementary Materials

The following supporting information can be downloaded at: https://www.mdpi.com/article/10.3390/ani16101441/s1, Table S1: Information of short-chain fatty acid standards used for LC–MS/MS analysis; Table S2: quality control results of samples used in this study.

## Abbreviations

The following abbreviations are used in this manuscript:

SCFAs	short-chain fatty acids
WF	grazing white goats
WB	barn-fed white goats
BF	grazing black goats
BB	barn-fed black goats
ORFs	Open reading frames
GH	glycoside hydrolases
GT	glycosyltransferases
CE	carbohydrate esterases
PL	polysaccharide lyases
CBM	carbohydrate-binding modules
